# Polygonati Rhizoma Polysaccharide Prolongs Lifespan and Healthspan in *Caenorhabditis elegans*

**DOI:** 10.3390/molecules28052235

**Published:** 2023-02-27

**Authors:** Yage Luan, Yu Jiang, Rong Huang, Xuan Wang, Xiujuan He, Yonggang Liu, Peng Tan

**Affiliations:** College of Chinese Medicine, Beijing University of Chinese Medicine, Beijing 102488, China

**Keywords:** Polygonati Rhizoma polysaccharide, lifespan extension, oxidative stress, insulin/IGF-1 signaling

## Abstract

Polygonati Rhizoma is the dried rhizome of *Polygonatum kingianum* coll.et hemsl., *Polygonatum sibiricum* Red. or *Polygonatum cyrtonema* Hua, and has a long history of medication. Raw Polygonati Rhizoma (RPR) numbs the tongue and stings the throat, while prepared Polygonati Rhizoma (PPR) can remove the numbness of the tongue, and at the same time enhance its functions of invigorating the spleen, moistening the lungs and tonifying the kidneys. There are many active ingredients in Polygonati Rhizoma (PR), among which polysaccharide is one of the most important active ingredients. Therefore, we studied the effect of Polygonati Rhizoma polysaccharide (PRP) on the lifespan of *Caenorhabditis elegans* (*C. elegans*) and found that polysaccharide in PPR (PPRP) was more effective than Polysaccharide in RPR (RPRP) in prolonging the lifespan of *C. elegans*, reducing the accumulation of lipofuscin, and increasing the frequency of pharyngeal pumping and movement. The further mechanism study found that PRP can improve the anti-oxidative stress ability of *C. elegans*, reduce the accumulation of reactive oxygen species (ROS) in *C. elegans*, and improve the activity of antioxidant enzymes. The results of quantitative real-time PCR(q-PCR) experiments suggested that PRP may prolong the lifespan of *C. elegans* by down-regulating d*af-2* and activating *daf-16* and *sod-3*, and the transgenic nematode experiments were consistent with its results, so it was hypothesized that the mechanism of age delaying effect of PRP was related to *daf-2*, *daf-16* and *sod-3* of the insulin signaling pathway. In short, our research results provide a new idea for the application and development of PRP.

## 1. Introduction

With the changes in diet, environment, and lifestyle in modern society, population aging has become an important social issue, and aging and age-related diseases have attracted increasing attention [1]. Aging is an inevitable physiological process with multifactorial interactions and is usually accompanied by a variety of diseases, such as metabolic diseases [2], cardiovascular diseases [3], and neurological diseases [4]. Therefore, how to extend life span, find anti-aging strategies, and improve health has become a research hot-spot.

Polygonati Rhizoma is the dried rhizome of *Polygonatum kingianum* coll.et hemsl., *Polygonatum sibiricum* Red. or *Polygonatum cyrtonema* Hua, and has a long history of medication. RPR numbs the tongue and stings the throat, while prepared PPR can remove the numbness of the tongue, and at the same time enhance its functions of invigorating the spleen, moistening the lungs, and tonifying the kidneys. There are many active ingredients in PR, including polysaccharides [5], saponins [6], flavonoids [7], and alkaloids [8], among which polysaccharide is one of the most important active ingredients.

Modern pharmacological studies have shown the wide range of pharmacological effects of PR, mainly in kidney protection, cardio protection, antioxidant, anti-Alzheimer’s disease, and anti-cancer [9]. For example, Shen et al. reported that PCP significantly increased superoxide dismutase (SOD) and glutathione peroxidase (GSH-Px) activity and decreased malondialdehyde (MDA) content in 3-, 6-, and 9-month-old mice, indicating that PCP can increase antioxidant enzyme activity to prevent lipid peroxidation and oxidative stress induced by forceful exercise [10]. Li et al. found that for cervical cancer He La cells, PCHPs could up-regulate the expression of apoptotic genes Bak, Cytc, Puma, and caspases-3 and related proteins, while down-regulating the expression of anti-apoptotic genes Bcl-2, Bcl-x L, and Bcl-2 proteins, thus promoting cancer cell apoptosis to exert anti-tumor effects [11].

Studies have shown that there are about 60~80% of human homologous genes in *C. elegans* genome, and it has the advantages of a short life cycle and easy operation, so *C. elegans* is an ideal biological model for anti-aging studies [12]. However, there have been no reports on the difference of anti-aging effect and the mechanism of the anti-aging effect of PRP before and after processing in *C. elegans*, so in this paper, we mainly studied the activity and mechanism of PRP in delaying senescence of *C. elegans*.

## 2. Results

### 2.1. Analysis of the Monosaccharide Composition of RPRP and PPRP

The ratio of mannose, rhamnose, glucose, galactose, and arabinose in RPRP and PPRP was determined by HPLC derivatization, the molar ratio of mannose: rhamnose: glucose: galactose: arabinose in RPRP was 0.18: 0.06: 1: 0.11: 0.07; the molar ratio of mannose: rhamnose: glucose: galactose: arabinose in PPRP was 0.97: 0.42: 1: 3.02: 0.67. The results showed that the content of galactose in polysaccharide increased after the concoction of PRP.

### 2.2. Effect of RPRP and PPRP on the Longevity of N2

**Figure 1:**
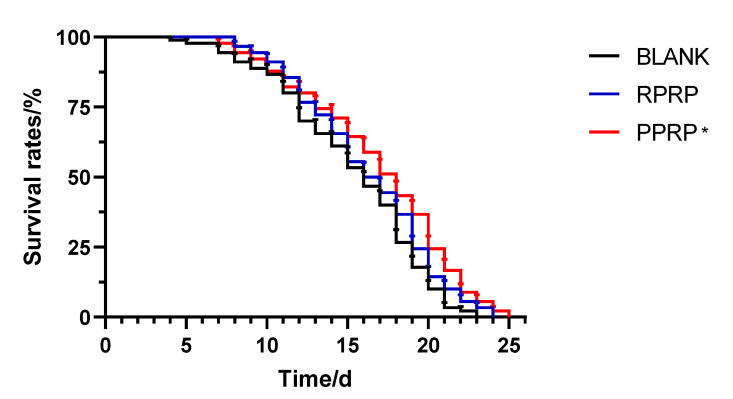
Effect of RPRP and PPRP on the longevity of N2. The number of dead nematodes was recorded every 24 h until all nematodes were dead and survival curves were plotted. * *p* < 0.05, compared to BLANK.

The experimental survival rate curve of the N2 lifespan is shown in Figure 1. Compared with the blank group, the survival rate of nematodes in the PPRP group was increased and the average lifespan was prolonged by 10.44%, the difference was statistically significant; compared with the blank group, although the RPRP group could prolong the maximum lifespan of nematodes, the average lifespan was prolonged by 6.20%, the difference was not significant (Table 1). It indicates that PPRP has a better effect of prolonging the lifespan of *C. elegans* than RPRP at this experimental concentration.

### 2.3. Effect of RPRP and PPRP on Lipofuscin Accumulation in N2

**Figure 2:**
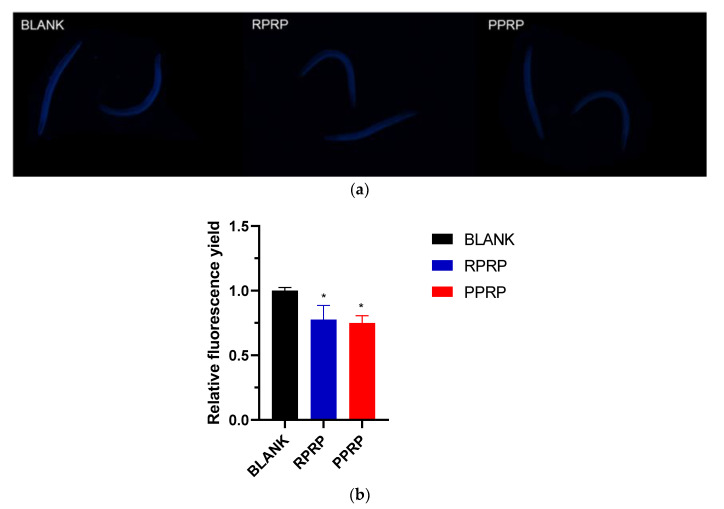
(**a**). Blue fluorescence produced by lipofuscin of N2 under fluorescence irradiation. Observation of lipofuscin accumulation levels in nematodes under a fluorescence microscope with an excitation wavelength of 340–380 nm. (**b**). Effect of RPRP and PPRP on lipofuscin accumulation in N2. Image J was used to measure the fluorescence intensity to indicate the amount of lipofuscin in nematodes, and the data were analyzed in terms of the fluorescence intensity of the experimental group relative to the blank group.* *p* < 0.05, compared to BLANK.

Lipofuscin is not eliminated by cytosolic action in *C. elegans* and accumulates in cells in an age-dependent manner, thus reflecting the aging status of nematodes, therefore the intestinal lipofuscin level of nematodes is an important marker of aging [13]. As shown in Figure 2a, the blue fluorescence of lipofuscin accumulated in nematodes under the fluorescence microscope. In Figure 2b, both RPRP and PPRP significantly reduced the accumulation of lipofuscin in nematodes compared with the blank group, but there was no significant difference in the lipofuscin fluorescence of nematodes between RPRP and PPRP.

### 2.4. Effect of RPRP and PPRP on Pharyngeal Pump Frequency and Locomotion Frequency of N2

In addition to lifespan, the healthspan has become an increasingly important parameter for evaluating resistance to aging [14]. Thus, we also examined the frequency of pharyngeal pumping and locomotion in nematodes.

**Figure 3:**
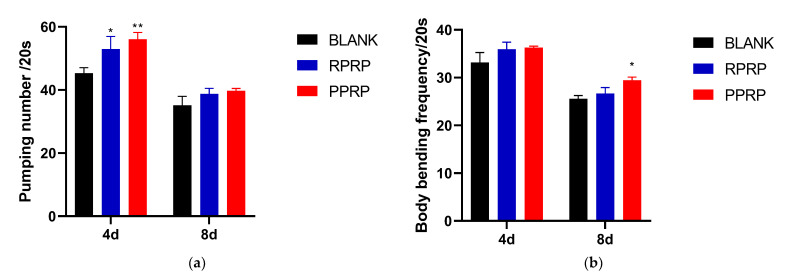
(**a**). Effect of RPRP and PPRP on the pharyngeal pumping rate of N2. Pharyngeal pump frequency of nematodes was measured on day 4 and day 8 of adult within 20 s. * *p* < 0.05, compared to BLANK; ** *p* < 0.01, compared to BLANK. (**b**). Effect of RPRP and PPRP on the f locomotion frequency of N2. The body bending frequency of nematodes was measured on day 4 and day 8 of adult within 20 s. * *p* < 0.05, compared to BLANK.

The experimental results of nematode pharyngeal pumping rate on day 4 and day 8 of adult worms are shown in Figure 3a. Compared with the blank group, on day 4 of adult nematodes, both RPRP and PPRP could improve the nematode pharyngeal pumping frequency, and the difference was statistically significant; on day 8 of adult nematodes, although RPRP and PPRP could also improve the nematode pharyngeal pumping frequency, the difference was not significant. It indicates that both RPRP and PPRP can reduce the senescence level of nematodes.

The nematode motility frequency was measured on day 4 and day 8 of adult nematodes. As shown in Figure 3b, the nematode locomotion frequency could be improved by PPRP on the 8th day of adult, and the difference was statistically significant; on the 4th day of adult, both RPRP and PPRP could improve the locomotion frequency of nematodes, but the difference was not significant. It indicates that the *C. elegans’* health cycle can be prolonged further by PPRP.

### 2.5. Effect of RPRP and PPRP on the Growth and Reproduction of N2

**Figure 4:**
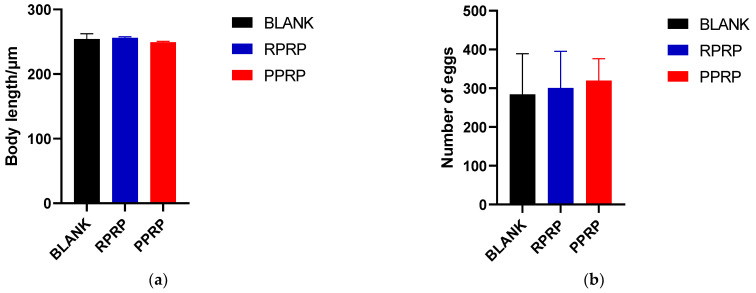
(**a**). Effect of RPRP and PPRP on the body length of N2. The nematodes were anesthetized and their body lengths were measured using a stereomicroscope. (**b**). Effect of RPRP and PPRP on egg production of N2. Egg production was recorded for each nematode every 24 h until the nematode stopped laying eggs.

As shown in Figure 4a, there was no significant difference in nematode body length in both RPRP and PPRP compared to the blank group. It is suggested that at this experimental concentration, RPRP and PPRP had no adverse effect on nematode growth.

The results of the nematode spawning experiment are shown in Figure 4b. Compared with the blank group, both RPRP and PPRP had no significant effect on the egg production of nematodes. It is suggested that this experimental concentration does not affect the normal reproduction of nematodes.

### 2.6. Effect of RPRP and PPRP on the Resistance to Oxidative Stress in N2

**Figure 5:**
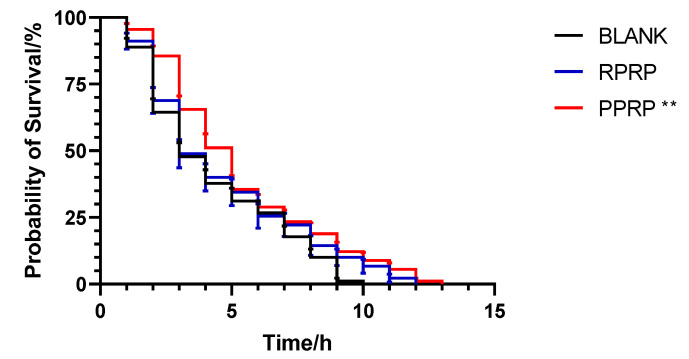
Effect of RPRP and PPRP on the resistance to oxidative stress in N2. The number of dead nematodes was recorded every 24 h until all nematodes were dead and survival curves were plotted. ** *p* < 0.01, compared to BLANK.

Juglone, as a pro-oxidant, causes oxidative stress and is considered a natural toxin [15]. Therefore, high concentrations of juglone are important for causing the rapid death of *C. elegans*, while the presence of antioxidants inhibits this effect [16]. The results of the antioxidant experiment are shown in Figure 5. Compared with the blank group, PPRP significantly improved the survival rate of nematodes in the oxidized environment of juglone, and the maximum lifespan of nematodes was extended by 3 h. The maximum lifespan of nematodes in RPRP was extended by 2 h, but the difference was not statistically significant. This indicates that the nematode resistance to oxidative stress is improved more by PPRP than RPRP.

### 2.7. Effect of RPRP and PPRP on Antioxidant Enzymes of N2

Antioxidant enzymes play an important role in reducing oxidative damage. SOD and CAT are two major antioxidant enzymes in nematodes that scavenge superoxide radicals that cause oxidative damage to biomolecules [17]. In order to study the effect of the polysaccharide of *C. elegans* on the activity of antioxidant enzymes in nematodes, the SOD and CAT activities were measured. As shown in Table 2, it was found that the PPRP could significantly increase the activity of SOD and CAT in *C. elegans*.

### 2.8. Effect of RPRP and PPRP on Reactive Oxygen Species (ROS) in N2

**Figure 6:**
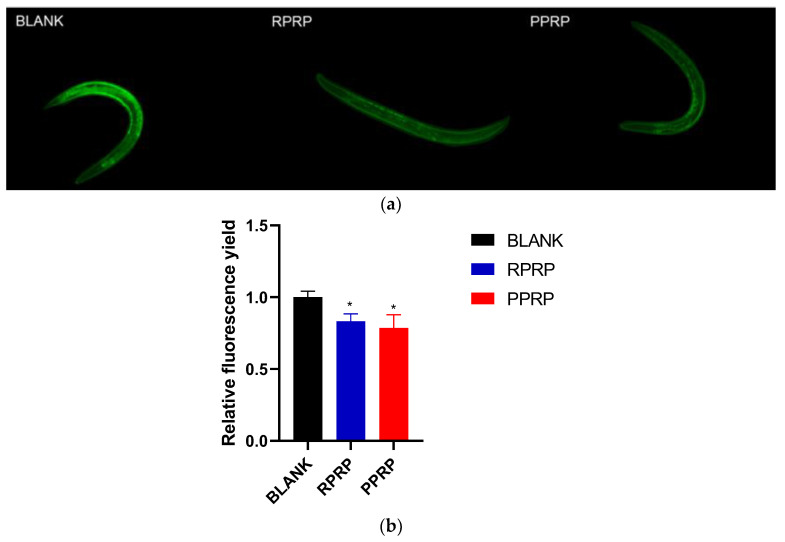
(**a**). Green fluorescence produced by ROS in N2 under fluorescence irradiation. ROS was determined by the fluorescent probe method. Adult nematodes were collected by adding 50 μmol/L of H2DCF-DA and incubated at 20 °C for 1 h. Nematode fluorescence was observed under a fluorescence microscope with excitation wavelengths of 485 nm. (**b**). Effect of RPRP and PPRP on ROS in N2. Image J was used to measure the fluorescence intensity, and the data were analyzed in terms of the fluorescence intensity of the experimental group relative to the blank group. * *p* < 0.05, compared to BLANK.

As nematodes age, their resistance to external stimuli decreases and reactive ROS increases, further accelerating the aging process and forming a vicious cycle [18]. In this study, we determined the effect of RPRP and PPRP on ROS in nematodes using the H2DCF-DA fluorescent probe method. The green fluorescence produced by ROS in nematodes after H2DCF-DA staining under fluorescence irradiation is shown in Figure 6a, and the experimental results are shown in Figure 6b. RPRP and PPRP could reduce ROS in nematodes compared to the blank group, and the difference was statistically significant. It indicates that the delay of nematode senescence by PRP may be related to the reduction of ROS levels in nematodes.

### 2.9. Effect of RPRP and PPRP on the Longevity of daf-16 Mutant

To explore whether the lifespan-prolonging effect of PRP was dependent on the longevity gene *daf-16* [19], the *daf-16* mutant strains CF1038 and DR26 were selected for lifespan experiments. The results are shown in Table 3 and Table 4. Compared with the blank group, RPRP and PPRP did not have any significant lifespan extension effect on CF1038 and DR26 transgenic nematodes, indicating that the *daf-16* is required for PRP to exert its anti-aging effect.

### 2.10. Effect of RPRP and PPRP on sod-3 Expression

**Figure 7:**
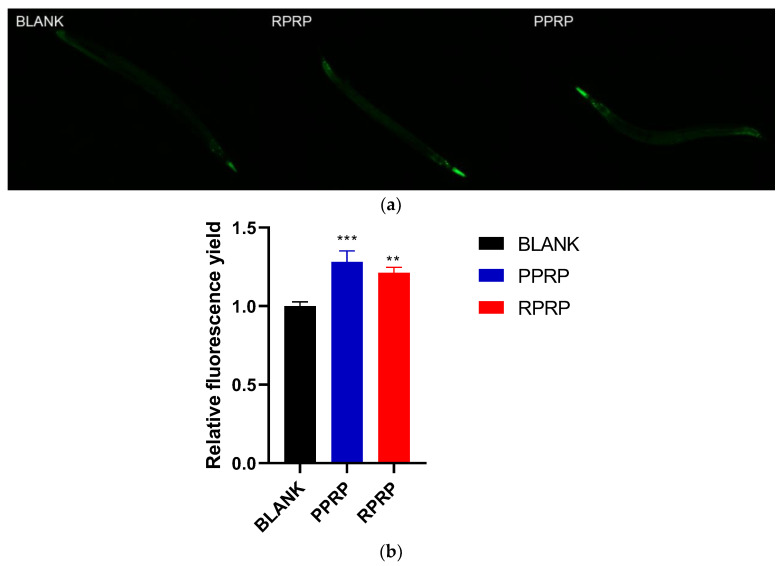
(**a**). Green fluorescence produced by CF1553 under fluorescence irradiation. Fluorescence of CF1553 was observed under a fluorescence microscope using an excitation wavelength of 485 nm. (**b**). Effect of RPRP and PPRP on *sod-3* expression. Image J was used to measure the fluorescence intensity, and the data were analyzed in terms of the fluorescence intensity of the experimental group relative to the blank group. *** *p* < 0.001, compared to BLANK; ** *p* < 0.01, compared to BLANK.

Typically, activation of *daf-16* subsequently leads to activation of other stress-responsive genes, such as *sod-3*, a key enzyme gene that protects nematodes from ROS [20]. Therefore, CF1553 (*sod-3*::GFP) transgenic nematodes were selected for the experiment, which showed green fluorescence around the head, tail, and around vulva under fluorescence microscopy, as shown in Figure 7a, and the experimental results were shown in Figure 7b. Compared with the blank group, both RPRP and PPRP increased the expression of *sod-3*::GFP in CF1553 transgenic nematodes, and it is hypothesized that the *sod-3* plays an important role in the delayed aging of *C. elegans*.

### 2.11. Effect of RPRP and PPRP on mRNA in N2

**Figure 8:**
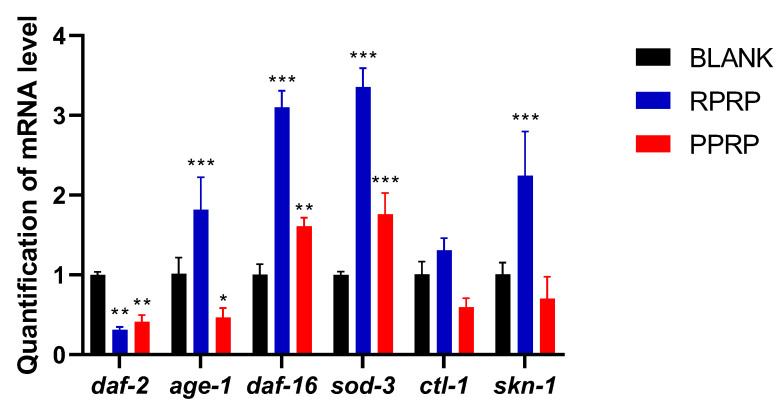
Effect of RPRP and PPRP on mRNA in N2. Determination of gene expression levels of *daf-2*, *age-1*, *daf-16*, *sod-3*, *ctl-1* and *skn-1* in N2 nematodes by RT-PCR. * *p* < 0.05, compared to BLANK; ** *p* < 0.01, compared to BLANK; *** *p* < 0.001, compared to BLANK.

RT-PCR was performed to detect the effects of RPRP and PPRP on the expression levels of *daf-2*, *age-1*, *daf-16*, *sod-3*, *ctl-1,* and *skn-1* mRNA. As shown in Figure 8, both RPRP and PPRP decreased the expression of *daf-2* and increased the expression of *daf-16* and *sod-3* in nematodes compared with the blank group, which verified that the RPRP and PPRP could increase the activity of SOD in the previous content and that RPRP and PPRP depend on *daf-16* to exert anti-aging effects.

### 2.12. Effect of RPRP and PPRP on the Longevity of daf-2 Mutant

In order to further verify whether the anti-aging effect exerted by PRP is related to *daf-2*, transgenic nematodes CB1370 were selected for the lifespan experiment, and the experimental results are shown in Table 5. Compared with the blank group, the average lifespan of nematodes in RPRP was extended by 3.21%, and the average lifespan of nematodes in PPRP was extended by 1.48%, which were not significantly different, further proving that PRP exerts anti-aging effects related to *daf-2*.

## 3. Discussion

PR is considered a “longevity and longevity medicine”, and the tonic effect is enhanced by wine processing, but there are few related studies. In this paper, we found that compared with the blank group, RPRP showed certain anti-aging potential, and PPRP showed a significant anti-aging effect, and the effect of PPRP was better than that of RPRP, which is consistent with the concoction principle of PR concoction for potency enhancement, and clarifies the scientific connotation of concoction from the perspective of aging.

*C. elegans* has the advantages of small size, ease of handling, short life cycle, detailed genetics and signaling pathways, high genetic conservation with human genes, and cost-effectiveness of high-throughput screening [21], making it an excellent model organism for studying aging in current studies. Therefore, in this paper, we chose various indicators to evaluate the anti-aging activity of PRP and found that PPRP significantly prolonged the lifespan of nematodes, reduced the accumulation of lipofuscin in nematodes, and delayed the aging of nematodes. With the prevalence of aging-related diseases, the healthspan has also become an essential parameter for assessing the anti-aging potential of drugs. Therefore, by measuring the pharyngeal pump and motility of adult nematodes, it was found that for the same period of nematodes, PPRP could significantly improve the health level of nematodes. In addition, the anti-aging effect of the drug should be accompanied by minimizing damage to the organism. Therefore, in this paper, we measured the effect of the PRP on the egg production and body length of nematodes, and the results showed that there was no adverse effect on the growth and development of *C. elegans* at this experimental concentration.

Currently, various theories propose different mechanisms of aging, including the free radical damage theory, the caloric restriction theory, and the telomere aging theory. Free radicals are continuously produced in the body along with metabolism and have a robust oxidative reaction capacity. The body has its own free radical scavenging systems, such as catalase, which can scavenge excess free radicals in the body and maintain the dynamic balance of free radicals. According to the theory of free radical damage, as aging occurs, the body cannot maintain the dynamic balance of free radical production and scavenging. Excessive free radicals can trigger lipid peroxidation in cell membranes and can also cause nucleic acid degeneration and dysfunction in cells, causing the body to develop towards aging [22]. In this paper, we simulated the peroxidation phenomenon generated by the increase of free radicals through oxidative stress experiments, and measured the effects of PRP on reactive oxygen species and antioxidant enzymes in nematodes. The results showed that PPRP could significantly improve the antioxidant property of nematodes, reduce the level of ROS in nematodes, and increase the activity of SOD and CAT, which also reflected the effect of PPRP that played a role in delaying the senescence of *C. elegans* by reducing oxidative damage.

*C. elegans*, one of the ideal models for studying the mechanism of drug-delayed aging, has been reported in more studies on its lifespan-related signaling pathways and is highly conserved in humans [23,24,25]. Among these signaling pathways, insulin/IGF-1 signaling (IIS) was the first one established for aging and was identified in Cryptobacterium hidradi through mutations in the *age-1* encoding phosphatidylinositol 3-kinase (PI3K) and mutations in the *daf-2* encoding the IGF-1 receptor [26], IIS is one of the key pathways known to regulate lifespan [27]. *Ctl-1* enables catalase activity and is predicted to be involved in the hydrogen peroxide catabolic process and response to hydrogen peroxide. *Sod-3* enables superoxide dismutase activity and is involved in the removal of superoxide radicals. *Skn-1* functions in the p38 MAPK pathway to regulate the oxidative stress response and in parallel to DAF-16/FOXO in the *daf-2* mediated insulin/IGF-1-like signaling pathway to regulate lifespan. It has been shown that the IIS pathway is involved in the lifespan-prolonging effect of saponins from bitter melon (BMS) under oxidative stress [28]. It has also been reported that Sonneradon A(SDA) prolongs nematode lifespan by affecting the upstream and downstream factors associated with *daf-16* in the IIS pathway [29]. In a lifespan experiment on a *daf-16* mutant strain, an important gene in the IIS, it was found that PRP requires the involvement of the d*af-16* to exert its anti-aging effect. Furthermore, *sod-3*, as the downstream gene of *daf-16*, is usually also readily activated, so the fluorescence of the *sod-3* mutant was measured, and the results showed that PRP could significantly increase the expression of *sod-3*. Next, RT-PCR was performed on IIS pathway-related genes, PRP was found to significantly decrease the expression of *daf-2* and increase the expression of *daf-16* and *sod-3*, which was consistent with the results of the *daf-16* transgenic nematode assay and the results of the *sod-3* fluorescence expression assay. To further verify the role of *daf-2*, a lifespan experiment of *daf-2* mutant strains was conducted, and the results showed that PRP delayed senescence in *C. elegans* in association with *daf-2*. In addition, we found that RPRP significantly reduced the accumulation of lipofuscin in nematodes, suggesting that RPRP has anti-aging activity, and combined with the results of mechanistic studies, we hypothesized that the age delaying effect of RPRP is related to *daf-2, daf-16* and *sod-3*. PPRP has significant effects on several indicators of anti-aging activity such as lifespan, lipofuscin, and body bending, and mechanistic studies found that the anti-aging effect of PPRP was also associated with *daf-2*, *daf-16,* and *sod-3*. Therefore, we suggest that PPRP has a better anti-aging effect, and further speculate that the anti-aging effect of PRP is associated with *daf-2*, *daf-16,* and *sod-3* in the IIS pathway.

## 4. Materials and Methods

### 4.1. Chemicals and Reagents

NKA-9 macroporous adsorption resin was purchased from Solarbio Co. (Beijing, China); agar powder, peptone, and tryptone were purchased from Beijing Auboxing Biotechnology Co., Ltd. (Beijing, China); reactive oxygen species (ROS) kit, SOD kit (batch no. 20220307), CAT visible light kit (batch no. 20220330), and total protein quantitative test kit (batch no. 20220416) were purchased from Nanjing JianCheng Bioengineering Institute (Nanjing, China).

### 4.2. C. elegans

The strains used in this study were wild-type N2; CB1370, *daf-2*(e1370) Ⅲ; DR26, *daf-16*(m26); CF1038, *daf-16*(mu86); CF1553, *sod-3*::GFP; TJ1052, *age-1*(hx546)Ⅱ(obtained from the Chinese Academy of Sciences).

### 4.3. Preparation of RPRP and PPRP

Using the wine stewing method to concoct PPR, we took the appropriate amount of RPR and added 20% of yellow wine, smothered for 6 h, placed in a stew pot, stewed for 10 h with water heating and then stewed for 8 h, cut thick slices and dried at 60 °C for 48 h.

Extraction of crude polysaccharide by water extraction and alcohol precipitation method, followed by Savage method to remove protein, NKA-9 macroporous resin to remove pigment, 3500Da dialysis bag to remove small molecule impurities, concentrated under reduced pressure and then lyophilized.

### 4.4. Analysis of The Monosaccharide Composition of RPRP and PPRP

The samples were subjected to acid hydrolysis and derivatization and then determined by HPLC. The chromatographic column was an Agilent HPLC column (4.6 × 250 mm, 5 μm); mobile phase: (A) acetonitrile-(B) 0.05 mol/L phosphate buffer solution; gradient elution: 0–10 min, 15–17% (A); 10–18.5 min, 17–22.5% (A); 18.5–20 min, 22.5–23.5% (A); 20–32 min, 23.5–30% (A); volume. 23.5% (A); 20–32 min, 23.5–30% (A); volume flow rate 0.8 mL/min; column temperature 30 °C; detection wavelength 250 nm; injection volume 10 μL.

### 4.5. Exposure Experiments

The NGM solid medium was prepared according to the literature method [30]. The blank group added OP50 E. coli bacterial suspension dropwise on the surface of the medium as food for nematodes, and the experimental group coated E. coli bacterial suspension containing 2 mg/mL of RPRP and PPRP on the surface of the medium as food, respectively.

### 4.6. Longevity Experiments

The number of dead nematodes was recorded every 24 h. The surviving nematodes were transferred to a new medium every 48 h until all nematodes were dead. The N2, CF1038, and DR26 nematodes were incubated at 20 °C, and the CB1370 nematodes were incubated at 16 °C.

### 4.7. Lipofuscin Experiment

On day 8 of N2 adulthood, nematodes were transferred to slides containing 2% agarose pads for filming, observed, and photographed under a fluorescent microscope using excitation wavelengths of 340~380 nm and emission wavelengths of 430 nm. Fluorescence intensity was measured using Image J software.

### 4.8. Pharyngeal Pump Assay and Locomotion Assay

The number of pharyngeal pumps and the number of nematodes doing sinusoidal movements in 20 s per nematode were measured on days 4 and 8 of N2 adulthood.

### 4.9. Body Length Experiment

Adults were transferred to 2% agarose pads, anesthetized and photographed with a body microscope to measure their body length.

### 4.10. Reproduction Experiments

One nematode in each medium was transferred every 24 h until the nematodes no longer laid eggs. The egg-laying medium was incubated at 20 °C for 2 d before counting the number of offspring.

### 4.11. Anti-oxidative Stress Assay

The adults were transferred to NGM medium containing 400 μM juglone and the number of surviving nematodes was counted every 1 h until all nematodes were dead.

### 4.12. Antioxidant Enzyme Assay

Approximately 1000 nematodes were collected from each medium and the experiments were carried out according to the kit instructions to determine SOD and CAT enzyme activities.

### 4.13. Determination of ROS

The nematodes were transferred to a slide containing 2% agarose pads, observed under a fluorescent microscope at 485 nm excitation wavelength and 530 nm emission wavelength, photographed and the fluorescence intensity was measured using Image J software.

### 4.14. sod-3 Fluorescence Expression Assay

Adult CF1553 was transferred to slides containing 2% agarose pads and photographed under a fluorescence microscope using excitation wavelength 485 nm and emission wavelength 530 nm, and then the fluorescence intensity was measured using Image J software.

### 4.15. RT-PCR Experiment

Approximately 1000 nematodes were collected into centrifuge tubes and rinsed 2–3 times. Total nematode RNA was extracted using an ultra-pure RNA extraction kit, and RNA concentration and purity were detected by UV absorption, RNA integrity was detected by denaturing agarose gel electrophoresis. The cDNA was synthesized by reverse transcription using the kit and then tested by RealTime PCR samples. Primer sequences for the genes of interest were as follows:

*daf-2*, F 5′-CGACTGAAGTGAATGGTGGA-3′, R 5′-CGCCGTTACTGAGACAAAATA-3′;
*age-1*, F 5′-CGCTGGCATCAAAATCTACA-3′, R 5′-ATTGGCAGTCGGTTCAGGAG-3′;
*daf-16*, F 5′-GGAGCCAAGAAGAGGATAAAGG-3′, R 5′-GGAGAAACACGAGACGACGAT-3′;
*sod-3*, F 5′-AGCATCATGCCACCTACGTGA-3′, R 5′-CACCACCATTGAATTTCAGCG-3′;
*ctl-1*, F 5′-TCCTACACGGACACGCATTAC-3′, R 5′-CGGAAACTGTTCGGGAAGTAA-3′;
*skn-1*, F 5′-TAGCCGACGACGAAGAAGAG-3′, R 5′-AGGTGTTGGACGATGGTGAA-3′;
actin, F 5′-GTCATGGTCGGTATGGGACA-3′, R 5′-TTGTAGAAGGTGTGATGCCAGA-3′.

### 4.16. Statistical Methods

Data were analyzed by SPSS 20.0 software, and the measurement data were expressed as mean ± standard deviation ($\bar{x}\;\pm \;$*s*), and one-way ANOVA was used, and GraphPad Prism 8 software was applied to make graphs. *p* < 0.05 was considered statistically significant.

## 5. Conclusions

Our studies show that RPRP significantly reduces the accumulation of lipofuscin in nematodes, PPRP significantly extends the lifespan of nematodes, reduces lipofuscin accumulation, and increases pharyngeal pump frequency and body bending frequency. We, therefore, consider that PPRP has better anti-aging activity than RPRP. Mechanistic studies have shown that PRP can improve the resistance to oxidative stress, reduce the accumulation of ROS in *C. elegans* and increase the activity of antioxidant enzymes. It was also found that the mechanism of PRP in delaying aging may be related to *daf-2*, *daf-16,* and *sod-3* of the IIS. In conclusion, our results suggest that PRP could be a promising natural anti-aging component for further research.

## Figures and Tables

**Table 1:** Effect of RPRP and PPRP on the longevity of N2 (*n* = 90,$\;\bar{x}\;\pm \;$ *s*).

Group	Maximum Lifespan/d	Average Lifespan/d
BLANK	23	15.32 ± 0.43
RPRP	24	16.27 ± 0.28
PPRP	25	16.92 ± 0.21 *

* *p* < 0.05, compared to BLANK.

**Table 2:** Effect of RPRP and PPRP on SOD and CAT enzymes of N2.

Group	SOD (U/mg)	CAT (U/mg)
BLANK	4.71 ± 0.86	2.54 ± 0.69
RPRP	8.10 ± 1.85 *	4.02 ± 0.52
PPRP	9.00 ± 1.86 *	5.41 ± 0.98 *

* *p* < 0.05, compared to BLANK.

**Table 3:** Effect of RPRP and PPRP on the longevity of CF1038 (*n* = 90,$\;\bar{x}\pm \;$ *s*).

Group	Maximum Lifespan/d	Average Lifespan/d
BLANK	20	13.61 ± 0.53
RPRP	20	14.14 ± 0.32
PPRP	20	14.23 ± 1.04

**Table 4:** Effect of RPRP and PPRP on the longevity of DR26 (*n* = 90,$\;\bar{x}\;\pm \;$ *s*).

Group	Maximum Lifespan/d	Average Lifespan/d
BLANK	21	14.40 ± 0.83
RPRP	22	15.09 ± 0.25
PPRP	22	15.09 ± 1.01

**Table 5:** Effect of RPRP and PPRP on the longevity of CB1370 (*n* = 90, $\bar{x}\pm \;$ *s*).

Group	Maximum Lifespan/d	Average Lifespan/d
BLANK	30	19.58 ± 1.16
RPRP	30	20.21 ± 0.11
PPRP	30	19.87 ± 0.27

## Data Availability

Data is contained within the article.

## Sample Availability

Samples of the compounds are not available from the authors.
